# The genome sequence of wood avens, *Geum urbanum *L., 1753

**DOI:** 10.12688/wellcomeopenres.19664.1

**Published:** 2023-08-30

**Authors:** Maarten J. M. Christenhusz, Meng Lu, Royal Botanic Gardens Kew Genome Acquisition Lab, Royal Botanic Garden Edinburgh Genome Acquisition Lab, Plant Genome Sizing collective, Darwin Tree of Life Barcoding collective, Wellcome Sanger Institute Tree of Life programme, Wellcome Sanger Institute Scientific Operations: DNA Pipelines collective, Tree of Life Core Informatics collective, Darwin Tree of Life Consortium

**Affiliations:** 1 Royal Botanic Gardens Kew, Richmond, England, UK; 2 The University of Edinburgh, Edinburgh, Scotland, UK; 3 Royal Botanic Garden Edinburgh, Edinburgh, Scotland, UK

**Keywords:** Geum urbanum, wood avens, genome sequence, chromosomal; Rosales

## Abstract

We present a genome assembly from an individual *Geum urbanum* (wood avens; Streptophyta; Magnoliopsida; Rosales; Rosaceae). The genome sequence is 1,304.9 megabases in span. Most of the assembly is scaffolded into 21 chromosomal pseudomolecules. The mitochondrial and plastid genomes have also been assembled and are 335.5 and 156.1 kilobases in length respectively.

## Species taxonomy

Eukaryota; Viridiplantae; Streptophyta; Embryophyta; Tracheophyta; Spermatophyta; Magnoliopsida; eudicotyledons; Gunneridae; Pentapetalae; rosids; fabids; Rosales; Rosaceae; Rosoideae; Colurieae; *Geum*; *Geum urbanum* (Linnaeus 1753) (NCBI:txid57919).

## Background

**Figure 1.**
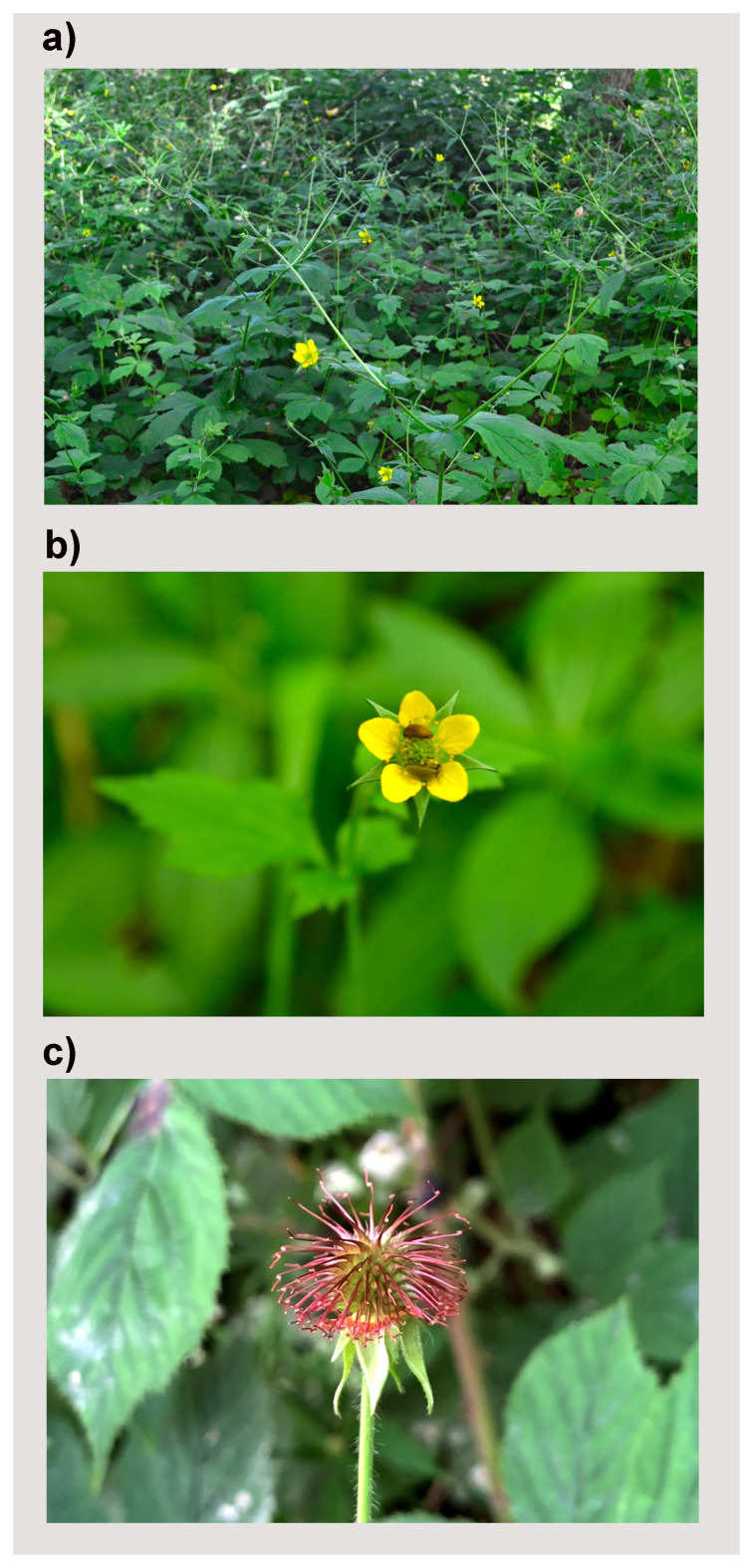
Ge *um urbanum* (not the sampled specimen) growing in secondary woodland. ( **a**) The plant habit with five-petal flowers in late May. ( **b**) Two small insects visiting the flower of *G. urbanum*. ( **c**) A fruiting head of *G. urbanum*. Photos taken by Meng Lu.

*Geum urbanum* L. (Rosaceae) is a widespread European perennial herb, the range of which extends to western Asia, western Siberia, and the northwest coast of Africa ( Taylor, 1997). It is native to Britain and Ireland and occurs abundantly, except in some parts of northern Scotland and Ireland ( Preston *et al.*, 2002; Stace *et al.*, 2019). Implied by its common name, wood avens, *G. urbanum* typically grows in woodland, shrubland, and hedgerows with well-drained conditions, but is also found in disturbed and more open habitats, waste grounds, gardens and parks ( Ruhsam *et al.*, 2011; Taylor, 1997). It is a predominantly self-pollinating species with the outcrossing rates ranging from 0.058 to 0.177 in natural populations ( Ruhsam *et al.*, 2010), yet its small, erect, yellow flowers can still attract pollinators ( Figure 1). The achene fruits of *G. urbanum* have a single hook ( Figure 1), which makes the seeds well-adapted to dispersal by animals ( Chen *et al.*, 2013; Gorb & Gorb, 2002; Smedmark & Eriksson, 2006).

Cytogenetic evidence shows that *G. urbanum* is an ancient hexaploid (2 *n* = 42) ( Gajewski, 1957; Gajewski, 1958), with molecular studies suggesting that allopolyploidisation gave rise to this hexaploid lineage in Rosoideae ( Gajewski, 1957; Smedmark *et al.*, 2005; Smedmark *et al.*, 2003). However, recent genetic studies show that this species largely behaves as a diploid, although with some additional duplicated gene copies ( Jordan *et al.*, 2018; Ruhsam, 2009). This species is known for its rampant hybridisation with a closely related species, *G. rivale*, where both occur in close proximity. These two species have several contrasting attributes, including mating system, flower morphology and habitat preference. Apart from its interesting biological features, many of the secondary metabolites of *G. urbanum* have important pharmacological uses ( Al-Snafi, 2019).

This genome will be extremely helpful for evolutionary studies aimed at understanding historical and contemporary hybridisation ( Jordan *et al.*, 2018; Ruhsam *et al.*, 2011) and the genetic basis of the selfing syndrome ( Sicard & Lenhard, 2011). It will also further contribute to uncovering the potential medical value of compounds produced by *G. urbanum*.

## Genome sequence report

The genome was sequenced from a *Geum urbanum* specimen collected from a garden bed at the Royal Botanic Gardens, Kew (latitude 51.48, longitude –0.30). Using flow cytometry, the genome size (1C-value) was estimated to be 1.64 pg, equivalent to 1,610 Mb. A total of 27-fold coverage in Pacific Biosciences single-molecule HiFi long reads and 64-fold coverage in 10X Genomics read clouds were generated. Primary assembly contigs were scaffolded with chromosome conformation Hi-C data. Manual assembly curation corrected 5 missing joins or mis-joins and removed one haplotypic duplication, reducing the scaffold number by 13.33%.

**Figure 2.**
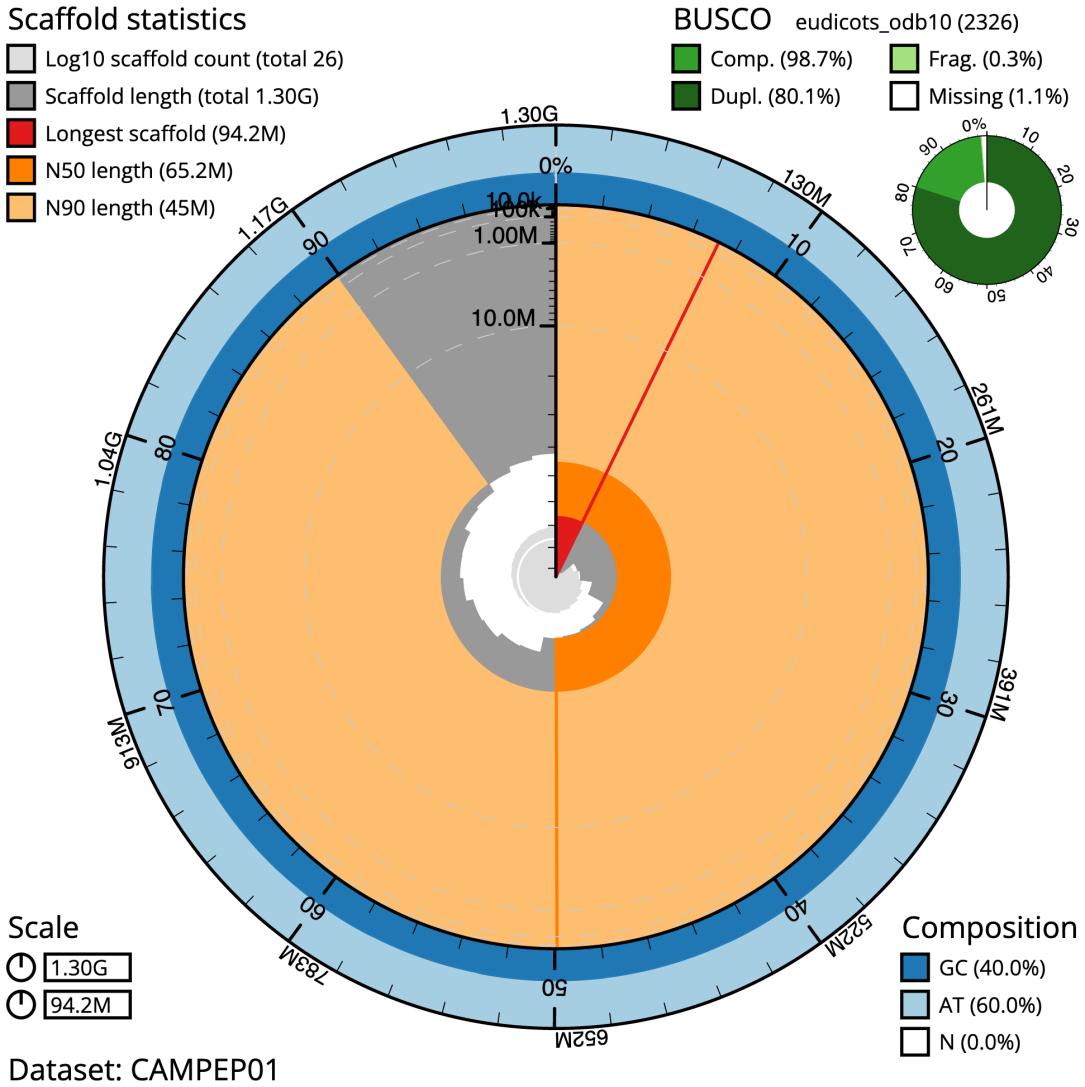
Genome assembly of *Geum urbanum*, drGeuUrba1.1: metrics. The BlobToolKit Snailplot shows N50 metrics and BUSCO gene completeness. The main plot is divided into 1,000 size-ordered bins around the circumference with each bin representing 0.1% of the 1,304,870,458 bp assembly. The distribution of scaffold lengths is shown in dark grey with the plot radius scaled to the longest scaffold present in the assembly (94,240,583 bp, shown in red). Orange and pale-orange arcs show the N50 and N90 scaffold lengths (65,224,196 and 44,983,829 bp), respectively. The pale grey spiral shows the cumulative scaffold count on a log scale with white scale lines showing successive orders of magnitude. The blue and pale-blue area around the outside of the plot shows the distribution of GC, AT and N percentages in the same bins as the inner plot. A summary of complete, fragmented, duplicated and missing BUSCO genes in the eudicots_odb10 set is shown in the top right. An interactive version of this figure is available at https://blobtoolkit.genomehubs.org/view/drGeuUrba1.1/dataset/CAMPEP01/snail.

The final assembly has a total length of 1,304.9 Mb in 26 sequence scaffolds with a scaffold N50 of 65.2 Mb ( Table 1). Most (99.95%) of the assembly sequence was assigned to 21 chromosomal-level scaffolds. Chromosome-scale scaffolds confirmed by the Hi-C data are named in order of size ( Figure 2– Figure 5; Table 2).

**Table 1.** Genome data for *Geum urbanum*, drGeuUrba1.1.

Project accession data		
Assembly identifier	drGeuUrba1.1	
Species	*Geum urbanum*	
Specimen	drGeuUrba1	
NCBI taxonomy ID	57919	
BioProject	PRJEB48840	
BioSample ID	SAMEA7522180	
Isolate information	leaf tissue; drGeuUrba1 – Monoecious	
Assembly metrics *		*Benchmark*
Consensus quality (QV)	59.6	*≥ 50*
*k*-mer completeness	99.99%	*≥ 95%*
BUSCO **	C:98.7%[S:18.6%,D:80.1%], F:0.3%,M:1.1%,n:2,326	*C ≥ 95%*
Percentage of assembly mapped to chromosomes	99.95%	*≥ 95%*
Sex chromosomes	Not applicable	*localised homologous pairs*
Organelles	Mitochondrial and plastid genomes assembled.	*complete single alleles*
Raw data accessions		
PacificBiosciences SEQUEL II	ERR7419408, ERR7419409	
10X Genomics Illumina	ERR7417841–ERR7417844	
Hi-C Illumina	ERR7417845	
PolyA RNA-Seq Illumina	ERR9435030	
Genome assembly		
Assembly accession	GCA_946800695.1	
*Accession of alternate haplotype*	GCA_946800285.1	
Span (Mb)	1,304.9	
Number of contigs	113	
Contig N50 length (Mb)	19.5	
Number of scaffolds	26	
Scaffold N50 length (Mb)	65.2	
Longest scaffold (Mb)	94.2	

* Assembly metric benchmarks are adapted from column VGP-2020 of “Table 1: Proposed standards and metrics for defining genome assembly quality” from ( Rhie *et al.*, 2021). ** BUSCO scores based on the eudicots_odb10 BUSCO set using v5.3.2. C = complete [S = single copy, D = duplicated], F = fragmented, M = missing, n = number of orthologues in comparison. A full set of BUSCO scores is available at https://blobtoolkit.genomehubs.org/view/drGeuUrba1.1/dataset/CAMPEP01/busco.

**Figure 3.**
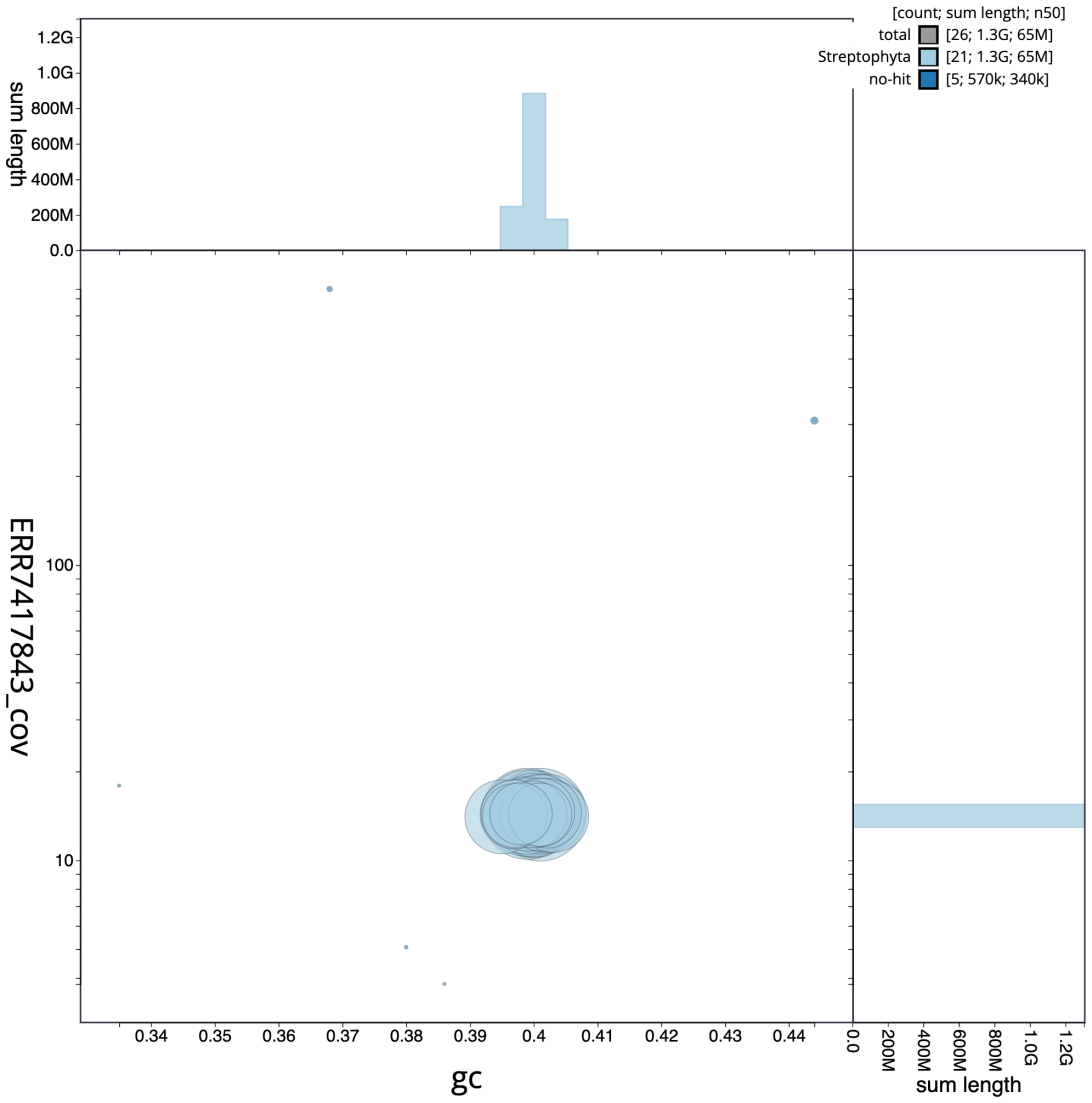
Genome assembly of *Geum urbanum*, drGeuUrba1.1: GC coverage. BlobToolKit GC-coverage plot. Scaffolds are coloured by phylum. Circles are sized in proportion to scaffold length. Histograms show the distribution of scaffold length sum along each axis. An interactive version of this figure is available at https://blobtoolkit.genomehubs.org/view/drGeuUrba1.1/dataset/CAMPEP01/blob.

**Figure 4.**
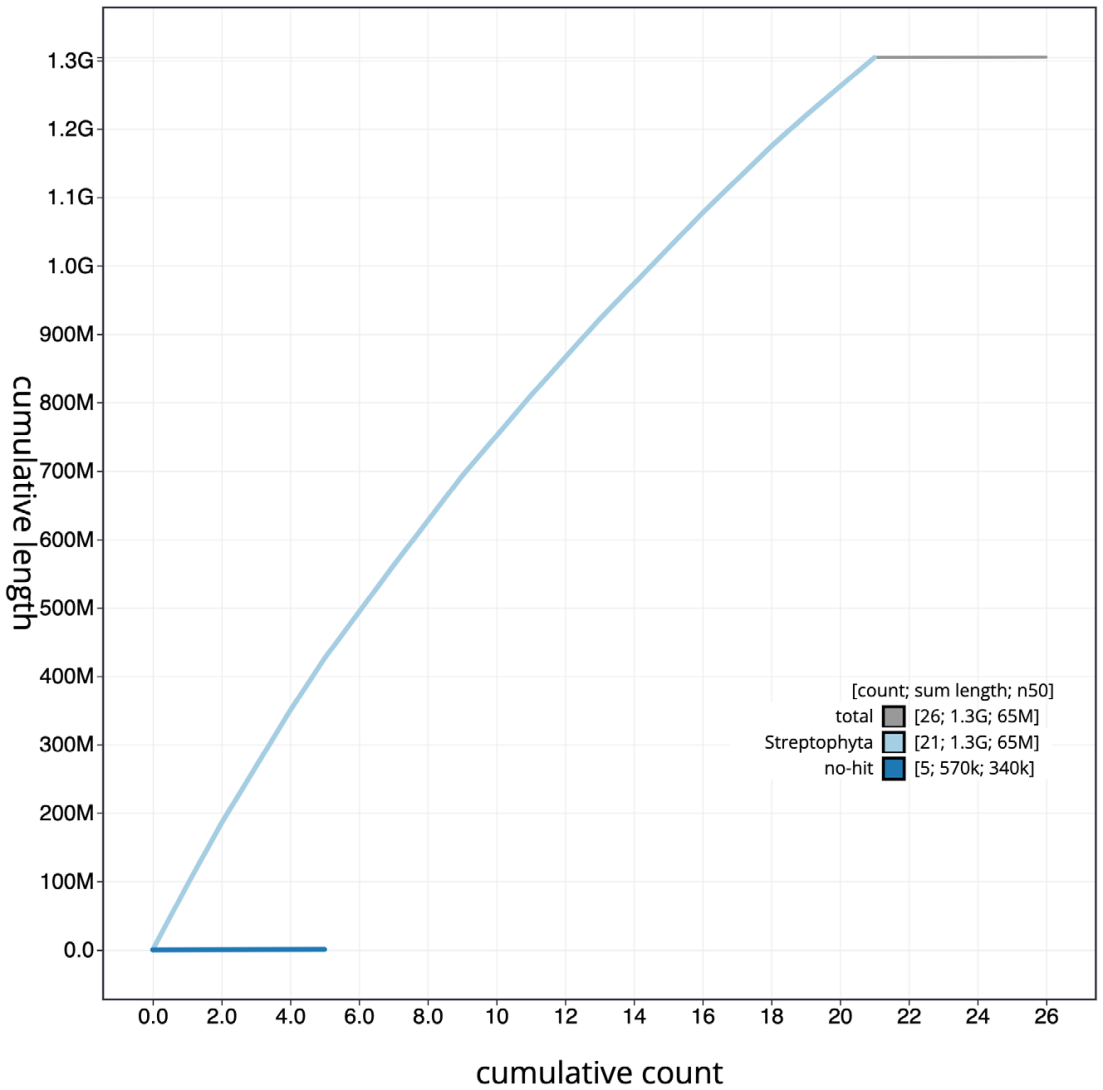
Genome assembly of *Geum urbanum*, drGeuUrba1.1: cumulative sequence. BlobToolKit cumulative sequence plot. The grey line shows cumulative length for all scaffolds. Coloured lines show cumulative lengths of scaffolds assigned to each phylum using the buscogenes taxrule. An interactive version of this figure is available at https://blobtoolkit.genomehubs.org/view/drGeuUrba1.1/dataset/CAMPEP01/cumulative.

**Figure 5.**
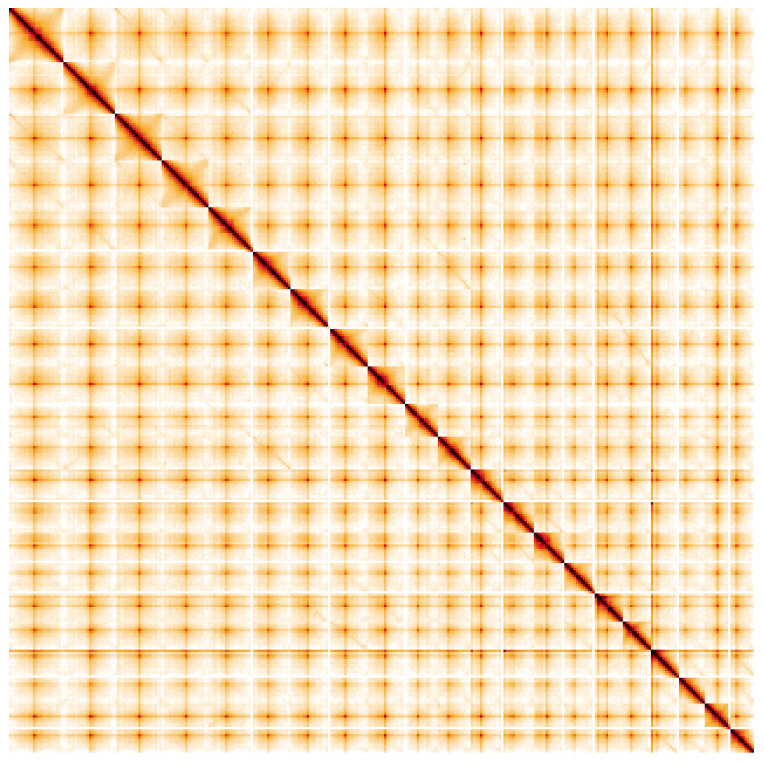
Genome assembly of *Geum urbanum*, drGeuUrba1.1: Hi-C contact map. Hi-C contact map of the drGeuUrba1.1 assembly, visualised using HiGlass. Chromosomes are shown in order of size from left to right and top to bottom. An interactive version of this figure may be viewed at https://genome-note-higlass.tol.sanger.ac.uk/l/?d=JSrsAc2aSfi-e9L51ilEkA.

**Table 2.** Chromosomal pseudomolecules in the genome assembly of *Geum urbanum*, drGeuUrba1.

INSDC accession	Chromosome	Size (Mb)	GC%
OX326997.1	1	94.24	40.1
OX326998.1	2	90.78	39.9
OX326999.1	3	82.87	40
OX327000.1	4	81.92	39.9
OX327001.1	5	76.26	40
OX327002.1	6	68.51	40.1
OX327003.1	7	66.65	40.2
OX327004.1	8	65.95	39.9
OX327005.1	9	65.22	40.1
OX327006.1	10	58.98	39.5
OX327007.1	11	58.47	39.9
OX327008.1	12	56.09	40.1
OX327009.1	13	55.44	40.3
OX327010.1	14	52.82	40.2
OX327011.1	15	51.58	39.7
OX327012.1	16	51.37	40.1
OX327013.1	17	48.95	39.7
OX327014.1	18	48.17	40
OX327015.1	19	44.98	39.7
OX327016.1	20	43.17	40.1
OX327017.1	21	41.87	39.8
OX327018.1	MT	0.34	44.4
OX327019.1	Pltd	0.16	36.8

The estimated Quality Value (QV) of the final assembly is 59.6 with *k*-mer completeness of 99.99%, and the assembly has a BUSCO v5.3.2 completeness of 98.7% (single = 18.6%, duplicated = 80.1%), using the eudicots_odb10 reference set ( *n* = 2,326).

Metadata for specimens, spectral estimates, sequencing runs, contaminants and pre-curation assembly statistics can be found at https://links.tol.sanger.ac.uk/species/57919.

## Methods

### Sample acquisition, genome size estimation and nucleic acid extraction

A specimen of *Geum urbanum* (drGeuUrba1) was collected from Bed 227 of the Rhododendron Dell at the Royal Botanic Gardens, Kew (latitude 51.48, longitude –0.30) on 26 August 2020. The specimen was picked by hand from weedy vegetation on the edge of the lawn by Maarten Christenhusz (Royal Botanic Gardens, Kew), collection number 9055. The specimen was identified based on its morphology by Maarten Christenhusz, and was preserved by freezing at –80°C.

Using flow cytometry, the genome size (1C-value) was estimated using the fluorochrome propidium iodide and following the ‘one-step’ method outlined in Pellicer *et al.* (2021). Specifically for this species, the General Purpose Buffer (GPB) supplemented with 3% PVP and 0.08% (v/v) beta-mercaptoethanol was used for isolation of nuclei ( Loureiro *et al.*, 2007), and the internal calibration standard was *Petroselinum crispum* ‘Champion Moss Curled’ with an assumed 1C-value of 2,200 Mb ( Obermayer *et al.*, 2002).

DNA was extracted at the Tree of Life laboratory, Wellcome Sanger Institute (WSI). The drGeuUrba1 sample was weighed and dissected on dry ice with tissue set aside for Hi-C sequencing. Leaf tissue was cryogenically disrupted to a fine powder using a Covaris cryoPREP Automated Dry Pulveriser, receiving multiple impacts. High molecular weight (HMW) DNA was extracted using the Illustra Nucleon PhytoPure HMW DNA extraction kit. HMW DNA was sheared into an average fragment size of 12–20 kb in a Megaruptor 3 system with speed setting 30. Sheared DNA was purified by solid-phase reversible immobilisation using AMPure PB beads with a 1.8× ratio of beads to sample to remove the shorter fragments and concentrate the DNA sample. The concentration of the sheared and purified DNA was assessed using a Nanodrop spectrophotometer and Qubit Fluorometer and Qubit dsDNA High Sensitivity Assay kit. Fragment size distribution was evaluated by running the sample on the FemtoPulse system.

RNA was extracted from leaf tissue of drGeuUrba1 in the Tree of Life Laboratory at the WSI using TRIzol, according to the manufacturer’s instructions. RNA was then eluted in 50 μl RNAse-free water and its concentration assessed using a Nanodrop spectrophotometer and Qubit Fluorometer using the Qubit RNA Broad-Range (BR) Assay kit. Analysis of the integrity of the RNA was done using Agilent RNA 6000 Pico Kit and Eukaryotic Total RNA assay.

### Sequencing

Pacific Biosciences HiFi circular consensus and 10X Genomics read cloud DNA sequencing libraries were constructed according to the manufacturers’ instructions. Poly(A) RNA-Seq libraries were constructed using the NEB Ultra II RNA Library Prep kit. DNA and RNA sequencing were performed by the Scientific Operations core at the WSI on Pacific Biosciences SEQUEL II (HiFi), Illumina HiSeq 4000 (RNA-Seq) and Illumina NovaSeq 6000 (10X) instruments. Hi-C data were also generated from leaf tissue of drGeuUrba1 using the Arima v2 kit and sequenced on the Illumina NovaSeq 6000 instrument.

### Genome assembly, curation and evaluation

Assembly was carried out with Hifiasm ( Cheng *et al.*, 2021) and haplotypic duplication was identified and removed with purge_dups ( Guan *et al.*, 2020). One round of polishing was performed by aligning 10X Genomics read data to the assembly with Long Ranger ALIGN, calling variants with FreeBayes ( Garrison & Marth, 2012). The assembly was then scaffolded with Hi-C data ( Rao *et al.*, 2014) using YaHS ( Zhou *et al.*, 2023). The assembly was checked for contamination and corrected as described previously ( Howe *et al.*, 2021). Manual curation was performed using HiGlass ( Kerpedjiev *et al.*, 2018) and Pretext ( Harry, 2022). The mitochondrial and chloroplast genomes were assembled using MBG ( Rautiainen & Marschall, 2021) from PacBio HiFi reads mapping to related genomes: a representative circular sequence was selected for each from the graph based on read coverage.

A Hi-C map for the final assembly was produced using bwa-mem2 ( Vasimuddin *et al.*, 2019) in the Cooler file format ( Abdennur & Mirny, 2020). To assess the assembly metrics, the k-mer completeness and QV consensus quality values were calculated in Merqury ( Rhie *et al*., 2020). This work was done using Nextflow ( Di Tommaso *et al.,* 2017) DSL2 pipelines “sanger-tol/readmapping” ( Surana *et al.,* 2023a) and “sanger-tol/genomenote” ( Surana *et al.*, 2023b). The genome was analysed within the BlobToolKit environment ( Challis *et al*., 2020) and BUSCO scores ( Manni *et al.*, 2021; Simão *et al.*, 2015) were calculated.

Table 3 contains a list of relevant software tool versions and sources.

**Table 3.** Software tools: versions and sources.

Software tool	Version	Source
BlobToolKit	4.0.7	https://github.com/blobtoolkit/blobtoolkit
BUSCO	5.3.2	https://gitlab.com/ezlab/busco
FreeBayes	1.3.1-17-gaa2ace8	https://github.com/freebayes/freebayes
Hifiasm	0.15.3	https://github.com/chhylp123/hifiasm
HiGlass	1.11.6	https://github.com/higlass/higlass
Long Ranger ALIGN	2.2.2	https://support.10xgenomics.com/genome-exome/ software/pipelines/latest/advanced/other-pipelines
MBG	-	https://github.com/maickrau/MBG
Merqury	MerquryFK	https://github.com/thegenemyers/MERQURY.FK
PretextView	0.2	https://github.com/wtsi-hpag/PretextView
purge_dups	1.2.3	https://github.com/dfguan/purge_dups
YaHS	1	https://github.com/c-zhou/yahs

### Wellcome Sanger Institute – Legal and Governance

The materials that have contributed to this genome note have been supplied by a Darwin Tree of Life Partner. The submission of materials by a Darwin Tree of Life Partner is subject to the **‘Darwin Tree of Life Project Sampling Code of Practice’**, which can be found in full on the Darwin Tree of Life website here. By agreeing with and signing up to the Sampling Code of Practice, the Darwin Tree of Life Partner agrees they will meet the legal and ethical requirements and standards set out within this document in respect of all samples acquired for, and supplied to, the Darwin Tree of Life Project. 

Further, the Wellcome Sanger Institute employs a process whereby due diligence is carried out proportionate to the nature of the materials themselves, and the circumstances under which they have been/are to be collected and provided for use. The purpose of this is to address and mitigate any potential legal and/or ethical implications of receipt and use of the materials as part of the research project, and to ensure that in doing so we align with best practice wherever possible. The overarching areas of consideration are:

•   Ethical review of provenance and sourcing of the material

•   Legality of collection, transfer and use (national and international) 

Each transfer of samples is further undertaken according to a Research Collaboration Agreement or Material Transfer Agreement entered into by the Darwin Tree of Life Partner, Genome Research Limited (operating as the Wellcome Sanger Institute), and in some circumstances other Darwin Tree of Life collaborators.

## Data Availability

European Nucleotide Archive: *Geum urbanum*. Accession number PRJEB48840; https://identifiers.org/ena.embl/PRJEB48840. ( Wellcome Sanger Institute, 2022) The genome sequence is released openly for reuse. The *Geum urbanum* genome sequencing initiative is part of the Darwin Tree of Life (DToL) project. All raw sequence data and the assembly have been deposited in INSDC databases. The genome will be annotated using available RNA-Seq data and presented through the Ensembl pipeline at the European Bioinformatics Institute. Raw data and assembly accession identifiers are reported in Table 1.

## Author information

Members of the Royal Botanic Gardens Kew Genome Acquisition Lab are listed here: https://doi.org/10.5281/zenodo.4786680.

Members of the Royal Botanic Garden Edinburgh Genome Acquisition Lab are listed here: https://doi.org/10.5281/zenodo.4786682.

Members of the Plant Genome Sizing collective are listed here: https://doi.org/10.5281/zenodo.7994306.

Members of the Darwin Tree of Life Barcoding collective are listed here: https://doi.org/10.5281/zenodo.4893703.

Members of the Wellcome Sanger Institute Tree of Life programme are listed here: https://doi.org/10.5281/zenodo.4783585.

Members of Wellcome Sanger Institute Scientific Operations: DNA Pipelines collective are listed here: https://doi.org/10.5281/zenodo.4790455.

Members of the Tree of Life Core Informatics collective are listed here: https://doi.org/10.5281/zenodo.5013541.
