# Supplementary material for: The genome sequence of wood avens, Geum urbanum L., 1753
Source: Wellcome Open Res. 2024 Jul 16;8:371. Originally published 2023 Aug 30. [Version 2] doi: 10.12688/wellcomeopenres.19664.2 (PMC11327653; doi:10.12688/wellcomeopenres.19664.2)
Supplement: Peer review history

# Reviewer report: Reviewer response for version 2

**DOI:** 10.21956/wellcomeopenres.25107.r90007

**Published:** 2024-09-01

**Authors:** Theivanayagam Maharajan

**Affiliations:** 1 Division of Plant Molecular Biology and Biotechnology, Department of Biosciences, Rajagiri College of Social Sciences, Cochin, Kerala, India

Accept the manuscript without further delay

Are sufficient details of methods and materials provided to allow replication by others?

No

Is the rationale for creating the dataset(s) clearly described?

Partly

Are the datasets clearly presented in a useable and accessible format?

No

Are the protocols appropriate and is the work technically sound?

Partly

Reviewer Expertise:

Functional Genomics and Genome Editing

I confirm that I have read this submission and believe that I have an appropriate level of expertise to confirm that it is of an acceptable scientific standard.

# Reviewer report: Reviewer response for version 2

**DOI:** 10.21956/wellcomeopenres.25107.r92345

**Published:** 2024-08-15

**Authors:** Abhisek Chakraborty

**Affiliations:** 1 Johannes Gutenberg University of Mainz, Mainz, Germany

1. The *Geum urbanum* genome presented in this data note is a high-quality reference standard genome based on the reported assembly statistics. However, since previous studies indicated towards the polyploidy of this species, the authors should report the LAI score to further strengthen the assembly statistics. Also, the BUSCO score for the coding gene set should be reported.

 2. Since the authors also assembled the mitochondrial and plastid genomes, these genome sequences should be deposited in a publicly available database. How many chloroplast and mitochondrial genes could be identified?

 3. The scripts used for the analyses are valuable to the research community and should be publicly available.

 4. In the abstract, “Most of the assembly” can be replaced with “99.95% of the assembly” for better readability.

Are sufficient details of methods and materials provided to allow replication by others?

Partly

Is the rationale for creating the dataset(s) clearly described?

Yes

Are the datasets clearly presented in a useable and accessible format?

Yes

Are the protocols appropriate and is the work technically sound?

Yes

Reviewer Expertise:

Genome assembly, Comparative Genomics, Evolutionary Genomics

I confirm that I have read this submission and believe that I have an appropriate level of expertise to confirm that it is of an acceptable scientific standard.

# Reviewer report: Reviewer response for version 1

**DOI:** 10.21956/wellcomeopenres.21781.r86519

**Published:** 2024-07-10

**Authors:** Theivanayagam Maharajan

**Affiliations:** 1 Division of Plant Molecular Biology and Biotechnology, Department of Biosciences, Rajagiri College of Social Sciences, Cochin, Kerala, India

Results should be described in detail because there is no information on how many genes, transcription factors, and markers have been identified from the genome of *Geum urbanum*. Why did the authors not submit the genome sequences at NCBI and phytozome databases? How many differentially expressed genes and proteins are identified from this study?  Hence, I advise the authors to please refer to already published articles and improve the contents of the manuscript.

Are sufficient details of methods and materials provided to allow replication by others?

No

Is the rationale for creating the dataset(s) clearly described?

Partly

Are the datasets clearly presented in a useable and accessible format?

No

Are the protocols appropriate and is the work technically sound?

Partly

Reviewer Expertise:

Functional Genomics and Genome Editing

I confirm that I have read this submission and believe that I have an appropriate level of expertise to state that I do not consider it to be of an acceptable scientific standard, for reasons outlined above.

# Response

**Published:** 2024-07-10

**Authors:** Tree of Life Team Sanger

**Affiliations:** Wellcome Trust Sanger Institute, UK

This data note reports on the whole genome assembly and its quality. This serves as a resource for researchers. It is not a research paper reporting on differentially expressed genes, transcription factors, and markers, although the resource is freely available to researchers who are studying the details of this species. The genome assembly is available on the NCBI ( GCA_946800695.1 ) as well as on ENA. We deposit the data on ENA, which is part of the INSDC, along with NCBI and the DNA DataBank of Japan (DDBJ). The INSDC's main policy is to provide permanent, free, and unrestricted access to all archived nucleotide data. The three organisations exchange data daily. Phytozome prioritises species sequenced at the Joint Genome Institute and selected species sequenced elsewhere and includes functional annotation. Since this genome assembly can be highly useful for researchers conducting functional annotation, we opted to deposit our data in the widely accessible INSDC databases. We believe that the current data note provides valuable information on the whole genome assembly and its quality, offering a crucial resource for further research. Given that our primary aim is to present the genome assembly rather than conduct a detailed functional annotation, we have ensured that the data is available through established and widely accessible platforms like NCBI and ENA. This approach aligns with our objective to make the genome data freely available to the research community. Since the first version of this data note, this *Geum urbanum* assembly has been annotated at the European Bioinformatics Institute (https://rapid.ensembl.org/Geum_urbanum_GCA_946800695.1/Info/Index), and we have updated the genome note in the second version to report on this annotation.

# Reviewer report: Reviewer response for version 1

**DOI:** 10.21956/wellcomeopenres.21781.r78942

**Published:** 2024-05-09

**Authors:** Zinian Wu

**Affiliations:** 1 Institute of Grassland Research, Chinese Academy of Agricultural Sciences, Hohhot, China

In this study, the high-quality genome of *Geum urbanum* was assembled. The Scaffold N50 was 65.2 MB, reaching the chromosome level. The estimated Quality Value ( QV ) of the final assembly is 59.6 with k-mer completeness of 99.99 %. and the assembly has a BUSCO v5.3.2 completeness of 98.7 %. It's a pretty perfect job. 

 Recommendations: 

1. LAI should be used to evaluate genome integrity. 
2. Complete the genome annotation and genome feature analysis as soon as possible.

Are sufficient details of methods and materials provided to allow replication by others?

Yes

Is the rationale for creating the dataset(s) clearly described?

Yes

Are the datasets clearly presented in a useable and accessible format?

Yes

Are the protocols appropriate and is the work technically sound?

Yes

Reviewer Expertise:

Identification and evaluation of plant germplasm resources

I confirm that I have read this submission and believe that I have an appropriate level of expertise to confirm that it is of an acceptable scientific standard.
